# The Role of Pharmacists and Community Pharmacies in the Screening, Knowledge, and Awareness of Diabetes Mellitus Type 2 in Jordanian People Visiting Community Pharmacies

**DOI:** 10.3390/jcm12030923

**Published:** 2023-01-24

**Authors:** Anas Khaleel, Mona Abu-Asal, Abdullah Bassam Zakariea, Rowan Alejielat, Anas Z. Al-Nweiran

**Affiliations:** Department of Pharmacology and Biomedical Sciences, Faculty of Pharmacy, Petra University, Amman 1196, Jordan

**Keywords:** type 2 diabetes mellitus, community pharmacy, screening, pharmacists, Jordan

## Abstract

Background: According to the International Diabetes Federation (IDF), diabetes is increasing exponentially worldwide and will become more prevalent than ever in the Middle East by 2045, with a 110% increase. This study aims to clarify the role of pharmacists and community pharmacies in the screening, knowledge, and awareness of Type 2 diabetes among Jordanian people who visit community pharmacies in Amman, Jordan. Methods: Study design: This was a cross-sectional prospective study that was conducted from September to December 2021 in Amman, Jordan. Data were collected using a standardized questionnaire that was composed of multiple parts. The first part collected information on demographics, residence, educational level, and insurance status; the second part was composed of 14 knowledge assessing questions; the last part was composed of the American Diabetes Association (ADA) diabetes risk score card test. Additionally, after confirming that each participant had returned their completed sheets, participants who scored greater than 5 had their blood sugar levels checked using a finger-prick blood test. The questionnaire was administered in person by a trained researcher. Using Slovin’s formula, a 95% confidence interval (CI), and a 0.05 margin of error, the sample size was determined to be 267 participants. The study included 305 participants. Descriptive and regression analyses were performed by using the Statistical Package for Social Science (SPSS) with a significance level of *p* < 0.05. Results: A significant relationship was found between specialty (medical education) and the knowledge of risk factors for Type 2 diabetes mellitus (T2DM), (*p* < 0.012). In terms of knowledge, from a total of 13 correct knowledge points (13 marks for correct answers out of 14), some subjects scored slightly higher than others (*n* = 175; 57.4% of participants scored above 7, nearly over half of the correct answers, compared to *n* = 130; 42.6% scoring below 7). We found 132 individuals (44%) with risk scores of five or above (high risk for developing T2DM according to ADA). Smokers comprised *n* = 138, 45%, and nonsmokers comprised *n* = 148, 48%. Although 50.5% of the participants (*n* = 154) held a bachelor’s, master’s, or doctorate degree, these degrees did not improve the participants’ overall general knowledge levels. The association was tested using chi-squared analysis, but no significance was found. Conclusions: Random visitors to Jordanian community pharmacies are expected to benefit from awareness and educational campaigns. These test results revealed a lack of knowledge, indicating the need for education to dispel myths and highlight the serious risks associated with T2DM. The study discovered that participants’ understanding of diabetes disease prevention through lifestyle and dietary changes was inadequate. A specialist-led educational program may increase knowledge among visitors who participate. In order to prevent the spread of diabetes, more campaigns and health-promoting and prevention educational activities are required.

## 1. Introduction

Diabetes mellitus (DM) is a chronic hyperglycemic condition caused by a group of metabolic disorders marked by a lack of insulin production, insulin activity, or both. Diabetes-induced hyperglycemia causes various long-term damage to many organs and tissues, particularly the eyes, kidneys, blood vessels, heart, and neuronal tissue [1]. It has become one of the top 10 global causes of death, with a 70% increase since 2000 [2]. Diabetes is classified into four general categories: Types 1 and 2 diabetes, gestational diabetes, and a particular type caused by other factors. However, Type 2 diabetes is the most common type, which accounts for 90–95% of all cases [3] and is significantly influenced by modifiable risk factors such as obesity [4], physical inactivity [5], smoking [6], and dietary factors [7], in addition to demographic determinants.

According to the International Diabetes Federation (IDF), in its 10th edition of the Diabetes Atlas in 2021, more than 1 out of every 10 adults globally now have diabetes, and this number will continue to rise rapidly [8]. Furthermore, diabetes is increasing exponentially worldwide, particularly in Africa and the Middle East, with a 156% increase in Africa and a 110% increase in the Middle East and North Africa by 2045, according to the IDF. The issue is that around 50% of those who have diabetes are completely unaware of their condition. In the early stages of diabetes, signs and symptoms are typically few; therefore, unnoticed symptoms persist until diabetic complications appear just before DM is diagnosed [9]. The reason for this rapid rise in diabetes rates is that the Middle Eastern population has one of the highest obesity rates in the world, which is the primary risk factor for DM and its complications [10,11]. This highlights the importance of diabetes screening in developing countries to detect undiagnosed DM patients or those with high-risk factors for developing DM. Furthermore, this is highly beneficial in establishing a prevention program for undiagnosed DM individuals to prevent this disease [12,13]. Jordan, a country in the Middle East, has one of the highest rates of obesity and smoking in the region. Surprisingly, significant DM mortality rates have not emerged in the Jordanian population [14]. Ajlouni et al. (2008) reported that the age-standardized prevalence of impaired fasting glucose (IFG) and diabetes was 7.8% and 17.1%, respectively, in Jordanians, with no statistically significant difference between men and women [15]. The American Diabetes Association (ADA) developed the diabetes risk test, a short questionnaire to aid in detecting both pre- and Type 2 diabetes. It consists of seven questions with a score range from 0 to 11 on age, gender, hypertension, physical activity, gestational diabetes, family history of diabetes, and obesity (using a height–weight chart to reference body mass index (BMI). This score assesses the risks of undiagnosed pre-DM patients and identifies undiagnosed DM patients at high risk [16].

A total of 13 risk factors associated with Type 2 diabetes mellitus (T2DM) from the literature include obesity, advances in age, males being more susceptible to diabetes than females, high blood pressure, the presence of diabetes in the father or mother or one of the siblings, physical inactivity, and females having gestational diabetes or having a baby weighing more than 4 kg. These are in addition to ethnic origins, decreased levels of cholesterol (HDL) or an increase in triglycerides, polycystic ovaries, depression, the presence of black pigmentation on the neck or under the armpits [17], and smoking [18]. There is a lack of knowledge about these risk factors among Jordanian people, and no previous studies have shown the role of pharmacists and community pharmacies in screening knowledge and awareness about T2DM. On the basis of the hypothesis that there is a lack of knowledge about the risk factors of T2DM, the aim of this study was to clarify the role of pharmacists and community pharmacies in the screening, knowledge, and awareness of Type 2 diabetes among Jordanian people who visit community pharmacies in Amman, Jordan.

## 2. Materials and Methods

### 2.1. Study Design

This was a cross-sectional prospective quantitative study that was conducted from September to December 2021 in multiple community pharmacies in Amman, Jordan. Data were collected using a standardized questionnaire. The structured questionnaire was composed of multiple parts. The first part collected qualitative information on demographics, residence, educational level, and insurance status, the second part was composed of 14 knowledge-assessing questions, and the last part was composed of the American Diabetes Association (ADA), Virginia, USA diabetes risk score card test. The questionnaire was administered in person by a trained researcher. Questionnaire development is explained in detail below. 

After ensuring that the participants had answered all questions and all sheets had been collected from the customers, the ADA diabetes risk scorecard test was used to calculate the individual risk of developing T2DM on a number scale explained below. Random blood sugar testing with a finger prick was performed on patients who had scored more than 5 (high-risk individuals) [19]. We followed the normal ADA range for random blood glucose (RBG): the criterion for normality is less than 140 mg/dL (7.8 mmol/L). More than 200 mg/dL (11.1 mmol/L) indicates diabetes, and between 140 and 199 mg/dL (7.8 mmol/L and 11.0 mmol/L) indicates prediabetes.

The study design was approved by the institutional review board (IBR) of the University of Petra (decision number: 202109009), and informed consent was obtained from all the participants. All participants were reminded that their participation was entirely voluntary and that they could opt out at any time.

### 2.2. Sample Size

The sample size was estimated for Amman’s nearly 4 million residents using Slovin’s formula [20], a 95% confidence interval (CI), and a 0.05 margin of error. Therefore, 267 people were needed in the sample to achieve the required confidence interval.

### 2.3. Characteristics of Participants

This study included 305 adults who lived in Amman, Jordan, and agreed to participate in the questionnaire. Study participants were chosen randomly from customers who had visited community pharmacies. 

#### 2.3.1. Inclusion Criteria

Participant over 18 who resided in Amman and visited community pharmacies.

#### 2.3.2. Exclusion Criteria

Patients who had been previously diagnosed with diabetes or prediabetes were excluded. When any participant had signs and symptoms of diabetes, they were also excluded.

### 2.4. American Diabetes Association Risk Scorecard

Seven questions were developed to determine the likelihood that the general public would develop diabetes [19]. The ADA previously published the current study’s questions, and their scores as follows:

1. Age: younger than 40 years (0 points), 40–49 years (1 point), 50–59 years (2 points), and 60 years or older (3 points).
2. Sex: male (1 point) and female (0 points).
3. History of gestational diabetes: yes (1 point) and no (0 points).
4. Family history of diabetes: yes (1 point) and no (0 points).
5. Hypertension history: yes (1 point) and no (0 points).
6. Physical activity: yes (0 points) and no (1 point).
7. Body weight: normal (0 points), overweight (1 point), obese (2 points), and extremely obese (3 points).

### 2.5. Instrument Development

A questionnaire was created and modified using information from the literature. The questionnaire was written in the Arabic language, translated in two ways, and consisted of two sections: demographics and knowledge. 

The demographics section included 9 questions regarding sex, age, marital status, geographic location, educational attainment, insurance status, smoking habits, income, and the presence of chronic disease (medications). 

The survey’s knowledge section included 14 questions that tested the respondents’ general knowledge of the risks of T2DM. Each item had a checkbox to select. One item was included to ensure that the participants could differentiate between intersex susceptibility to T2DM: females are more likely than males to develop diabetes mellitus (wrong answer). Correct: males are more likely than males to develop diabetes mellitus. The sum of correct answers was used to calculate the scores, with 13 being the highest possible score value.

A cut-off value for knowledge level was used to distinguish between poor and high knowledge. A total of 13 risk factors from the literature were used as knowledge questions (obesity, increase in age, males being more susceptible to diabetes than females, high blood pressure, the presence of diabetes in the father, mother, or one of the siblings, physical inactivity, having gestational diabetes or having a baby weighing more than 4 kg, ethnic origins, decreased level of cholesterol (HDL) or an increase in triglycerides, polycystic ovaries, depression, the presence of black pigmentation on the neck or under the armpits [17], and smoking [18]). We used roughly half of these factors (7/13) to represent knowledge among the participants. If a participant had scored 7 or higher, they were considered to be knowledgeable, whereas if they had scored less than 7, they were considered to be less knowledgeable. These values were statistically tested for associations among all variables. The Pearson chi-squared test was used for the categorical variables (including knowledge and demographics).

The validity and reliability of the questionnaires were not tested. All statistical analyses were carried out using the Statistical Package for Social Science (SPSS) (version 25.0, SPSS, Chicago, IL, USA) with a significance level of *p* < 0.05. All completed questionnaires were reviewed, and frequencies were calculated following the results. The factors influencing the knowledge of the Jordanian participants visiting community pharmacies were investigated using a multivariate logistic regression model.

## 3. Results

### 3.1. Demographics of Participants

Out of 315 completed questionnaires, 10 (3.2%; response rate = 97%) were excluded from the study because the respondents chose not to continue the interview. The capital of Amman received 96% of the location responses. A total of 132 (~44%) of the 315 participants recorded ADA risk scores of 5 and above and had their blood drawn (using a finger-prick blood test). 

In our sample, male participants comprised 54.1% (*n* = 165) of the total, while female participants comprised 45.9% (*n* = 140), and most attendees were married (*n* = 214, 70.2%). Participants under 40 years old were *n* = 115, 37.7%, while those over 40 years old were *n* = 190, 62.3%. The number of smoker participants in our sample was 138 (45%), and 148 (48%) were nonsmokers. About half of the participants (*n* = 151, 49.5%) had degrees equivalent to or lower than diplomas, while the other half (*n* = 154, 50.5%) held bachelor’s, master’s, or Ph.D. degrees. Moreover, we found that most of the participants (*n* = 256, 83%) did not have a medical degree, and only a small percentage of pharmacy visitors (*n* = 49, 16.1%) did. 

The statistical analysis of the collected data shows that 177 (58%) of the participants did not have any chronic diseases, whereas the remainder (*n* = 128, 42.0%) had at least one chronic condition such as hypertension. Moreover, we found that nearly two-thirds of participants (*n* = 208, 62%) did not have insurance, while only one-third (*n* = 97, 31%) did. 

When participants were asked about their monthly income, nearly 40% of participants refused to reveal their earnings. For the rest of the participants, the monthly salary incomes were almost evenly distributed above or below JOD 500, except for those with more than JOD 750 and less than JOD 1000, with a percentage of 3.3% (*n* = 10). Table 1 below summarizes the general information of the participants in the survey.

### 3.2. Prediabetics and Diabetics Were Identified due to the Community Pharmacy Screening Program

After testing all participants who had recorded an ADA score of 5 or higher, we found that 21 (7.3%) of the 305 participants had random blood sugar levels higher than 140 mg/dL but lower than 200 mg/dL. We also found five people with dangerously undiagnosed blood sugar levels above 200 mg/dL (range: 212–226 mg/dL). Such findings demonstrate how essential community pharmacies and pharmacists are in detecting T2DM.

The multivariate logistic regression results reveal a significant positive correlation (*p* < 0.05) between participants’ knowledge and the variables of type of college degree of participants and the salary of participants, as shown in Table 2 below.

## 4. Discussion

This study showed the role of pharmacists and community pharmacies in educating and discovering the risk factors of random visitors to local pharmacies in Amman. These test results revealed a lack of knowledge, and a significant number of individuals had a high risk to develop diabetes (~44%). 

Furthermore, the obtained results show a significant correlation (*p* < 0.012) between the educational type of the study (medical) and the level of knowledge of the risk factors for T2DM. These findings revealed that knowledge was below average, indicating a need for educational intervention to clarify misconceptions and highlight the severe risks of T2DM. According to the participants’ knowledge results, *n* = 175, 57.4% scored less than 7, and *n* = 130, 42.6% scored more than 7. 

Although more than half of the participants (*n* = 154, 50.5%) had bachelor’s, master’s, and Ph.D. degrees, these levels of education did not enhance their knowledge of DM disease. Furthermore, when categorical variables (including demographics and expertise) were evaluated, no significant results were found. In line with our findings, Lima et al. (2015) reported in a Brazilian study that diabetic elderly Brazilians with low education are nearly eight times more likely to have poor knowledge of DM than those with higher education [21]. Furthermore, Hu et al. [22] previously demonstrated these relationships in a Chinese study published in 2022. A similar Swedish study discovered that having a high educational level is generally a risk factor for diabetes [23]. This study revealed that participant knowledge level is unaffected by income or smoking status. There was no correlation between these factors (income and smoking) and knowledge of the risks of developing DM disease after testing them. In contrast to our findings, other researchers found that a low income caused many diabetic patients to experience diabetes distress, poor adherence to DM drugs, and uncontrolled blood sugar levels [24]. The authors showed that low-income areas had more DM and obesity [25].

Almost half of our participants were smokers (*n* = 138, 45.2%), which reflects very high smoking rates, agreeing with a previous finding that showed a high prevalence of smoking in Jordan [26,27,28,29]. In a recent report, the Kingdom of Jordan was classified as the sixth internationally: smoking prevalence was almost 70% between males, and approximately 10% among females [30]. This risk factor was recently added to the list of risk factors for diabetes mellitus [31]. Another report confirmed this causal relationship between smoking and T2DM from Japan [32]. Another significant report highlighted that quitting smoking is crucial not just for preventing macrovascular problems in diabetes, but also for minimizing microvascular damage, and may help with glycemic control in this condition [33], confirming relations between smoking and DM complications [34].

Nearly 62% of our cohort participants were over 40 years old (*n* = 190, 62.3%); this is considered to be the starting point for risk for developing T2DM [35]. We identified 132 individuals with risk scores of 5 and above (~44%), which showed high risks among random community pharmacy visitors and agreed with national reports that confirmed a high rate of prediabetics among Jordanians [36].

## 5. Limitations of the Study

The short duration of this study was a significant limitation. There was no follow-up monitoring to see if improved knowledge and daily activities affected long-term outcomes. Furthermore, because we only screened individuals in the Amman governorate, and we cannot generalize our findings to the rest of Jordan. Moreover, the sample of the study was generally small, and 40% of the participants refused to reveal their monthly income.

## 6. Conclusions

The role of pharmacists and community pharmacies in educating and discovering the risk factors of random visitors to local pharmacies in Amman was highlighted and revealed in this study. This study discovered that participants’ understanding of DM disease prevention through lifestyle and dietary changes was inadequate. Additional educational initiatives by a specialist may raise participants’ knowledge levels. Additional campaigns and health-promoting prevention education programs are required to continue preventing DM disease.

## Figures and Tables

**Table 1:** General information of the participants (*n* = 305).

Questions	Sample *n* (%)
Sex	
Female	140 (45.9)
Male	165 (54.1)
Age (years)	
≤40	115 (37.7)
>40	190 (62.3)
Education level	
Diploma or lower	151 (49.5)
Higher than diploma (bachelor’s, master’s, or Ph.D.)	154 (50.5)
Medical major degree	
Not having a medical degree	256 (83.9)
Having a medical degree	49 (16.1)
Income (Jordanian Dinar (JOD))	
Less than 250	25 (8.2)
251–500	81 (26.6)
501–750	31 (10.2)
751–1000	10 (3.3)
More than 1000	39 (12.8)
Not willing to disclose salary	119 (39.0)
Existence of health insurance	
Not having medical insurance	208 (68.2)
Having medical insurance	97 (31.8)
Marital status	
Married	214 (70.2)
Single	71 (23.3)
Divorced/widowed	20 (6.6)
Smoking habits	
Smoker	138 (45.2)
Quit smoking	16 (5.2)
Nonsmoker	148 (48.5)
Existence of chronic diseases	
Not having chronic diseases	177 (58.0)
Having chronic diseases	128 (42.0)

All variables were tested according to the knowledge level using a cut-off value of 7. Results revealed that only medical major participants had significantly more knowledge than that of nonmedical major participants (*p* < 0.012).

**Table 2:** Overall logistic regression analysis (multivariate) to evaluate predictors related to participants’ knowledge.

Independent Factors	Multivariate	
	Beta	*p*-Value
Age		
≤40 years		
>40 years	0.062	0.326
Sex		
Female		
Male	−0.038	0.531
Educational level		
Diploma or lower		
Higher than diploma (bachelor’s, master’s, or Ph.D.)	0.059	0.335
Medical Degree		
Not having a medical degree		
Having a medical degree	0.198	0.017
Salary		
≤500		
>500	−0.037	0.018
Smoking habits		
Smoker		
Nonsmoker	0.047	0.113

## Data Availability

Data is unavailable due to privacy or ethical restrictions.

## References

[1] American Diabetes Association (2013). Diagnosis and Classification of Diabetes Mellitus. Diabetes Care.

[2] World Health Organization The Top 10 Causes of Death. 9 December 2020. https://www.who.int/en/news-room/fact-sheets/detail/the-top-10-causes-of-death.

[3] American Diabetes Association (2019). 2. Classification and Diagnosis of Diabetes: Standards of Medical Care in Diabetes-2019. Diabetes Care.

[4] World Health Organization (2021). Obesity and Overweight. https://www.who.int/en/news-room/fact-sheets/detail/obesity-and-overweight.

[5] Malik V.S., Willett W.C., Hu F.B. (2013). Global obesity: Trends, risk factors and policy implications. Nat. Rev. Endocrinol..

[6] Pan A., Wang Y., Talaei M., Hu F.B., Wu T. (2015). Relation of active, passive, and quitting smoking with incident type 2 diabetes: A systematic review and meta-analysis. Lancet Diabetes Endocrinol..

[7] Ojo O. (2019). Dietary Intake and Type 2 Diabetes. Nutrients.

[8] Sun H., Saeedi P., Karuranga S., Pinkepank M., Ogurtsova K., Duncan B.B., Stein C., Basit A., Chan J.C.N., Mbanya J.C. (2022). IDF Diabetes Atlas: Global, regional and country-level diabetes prevalence estimates for 2021 and projections for 2045. Diabetes Res. Clin. Pract..

[9] Glovaci D., Fan W., Wong N.D. (2019). Epidemiology of Diabetes Mellitus and Cardiovascular Disease. Curr. Cardiol. Rep..

[10] Kilpi F., Webber L., Musaigner A., Aitsi-Selmi A., Marsh T., Rtveladze K., McPherson K., Brown M. (2014). Alarming predictions for obesity and non-communicable diseases in the Middle East. Public Health Nutr..

[11] Al-Shudifat A.E., Al-Shdaifat A., Al-Abdouh A.A., Aburoman M.I., Otoum S.M., Sweedan A.G., Khrais I., Abdel-Hafez I.H., Johannessen A. (2017). Diabetes Risk Score in a Young Student Population in Jordan: A Cross-Sectional Study. J. Diabetes Res..

[12] Echouffo-Tcheugui J.B., Mayige M., Ogbera A.O., Sobngwi E., Kengne A.P. (2012). Screening for hyperglycemia in the developing world: Rationale, challenges and opportunities. Diabetes Res. Clin. Pract..

[13] Hagstrom B., Mattsson B. (2002). Screening for diabetes in general practice. Opportunistic screening for diabetes in general practice is better than nothing. BMJ.

[14] Mayyas F.A., Ibrahim K.S. (2021). Predictors of mortality among patients with type 2 diabetes in Jordan. BMC Endocr. Disord..

[15] Ajlouni K., Khader Y.S., Batieha A., Ajlouni H., El-Khateeb M. (2008). An increase in prevalence of diabetes mellitus in Jordan over 10 years. J. Diabetes Complicat..

[16] Poltavskiy E., Kim D.J., Bang H. (2016). Comparison of screening scores for diabetes and prediabetes. Diabetes Res. Clin. Pract..

[17] American Diabetes Association Professional Practice Committee (2022). 2. Classification and Diagnosis of Diabetes: Standards of Medical Care in Diabetes-2022. Diabetes Care.

[18] U.S. Department of Health and Human Services (USDHHS) (2014). Smoking and Diabetes Factsheet. https://www.cdc.gov/tobacco/data_statistics/sgr/50th-anniversary/pdfs/fs_smoking_diabetes_508.pdf.

[19] Bang H., Edwards A.M., Bomback A.S., Ballantyne C.M., Brillon D., Callahan M.A., Teutsch S.M., Mushlin A.I., Kern L.M. (2009). Development and validation of a patient self-assessment score for diabetes risk. Ann. Intern. Med..

[20] Slovin E. (1960). Slovin’s Formula for Sampling Technique. https://prudencexd.weebly.com/.

[21] Lima A.P.D., Benedetti T.R.B., Rech C.R., Cardoso F.B., Portella M.R. (2020). Conhecimento e atitude sobre a diatebes tipo 2 em idosos: Estudo de base populacional. Ciência Saúde Coletiva.

[22] Hu J., Gruber K.J., Liu H., Zhao H., Garcia A.A. (2013). Diabetes knowledge among older adults with diabetes in Beijing, China. J. Clin. Nurs..

[23] Agardh E.E., Sidorchuk A., Hallqvist J., Ljung R., Peterson S., Moradi T., Allebeck P. (2011). Burden of type 2 diabetes attributed to lower educational levels in Sweden. Popul. Health Metr..

[24] Silverman J., Krieger J., Kiefer M., Hebert P., Robinson J., Nelson K. (2015). The relationship between food insecurity and depression, diabetes distress and medication adherence among low-income patients with poorly-controlled diabetes. J. Gen. Intern. Med..

[25] Gittelsohn J., Trude A. (2017). Diabetes and obesity prevention: Changing the food environment in low-income settings. Nutr. Rev..

[26] Burki T.K. (2019). Tobacco control in Jordan. Lancet Respir. Med..

[27] Bader R.K., Shihab R.A., Al-Rimawi D.H., Hawari F.I. (2017). Informing tobacco control policy in Jordan: Assessing the effectiveness of pictorial warning labels on cigarette packs. BMC Public Health.

[28] Shishani K., Nawafleh H., Jarrah S., Froelicher E.S. (2011). Smoking patterns among Jordanian health professionals: A study about the impediments to tobacco control in Jordan. Eur. J. Cardiovasc. Nurs..

[29] Al-Balas H., Al-Balas M., Al-Balas H.I., Khamees A., Talafha M., Nuseir A. (2021). Electronic Smoking Behavior Among Adult Males in Jordan. J. Commun. Health.

[30] Al-Tammemi A.B., Barakat M., Al Tamimi D., Alhallaq S.A., Al Hasan D.M., Khasawneh G.M., Naqera K.A., Jaradat R.M., Farah F.W., Al-Maqableh H.O. (2021). Beliefs Toward Smoking and COVID-19, and the Pandemic Impact on Smoking Behavior and Quit Intention: Findings from a Community-Based Cross-Sectional Study in Jordan. Tob Use Insights.

[31] Maddatu J., Anderson-Baucum E., Evans-Molina C. (2017). Smoking and the risk of type 2 diabetes. Transl. Res..

[32] Akter S., Goto A., Mizoue T. (2017). Smoking and the risk of type 2 diabetes in Japan: A systematic review and meta-analysis. J. Epidemiol..

[33] Sliwinska-Mosson M., Milnerowicz H. (2017). The impact of smoking on the development of diabetes and its complications. Diabetes Vasc. Dis. Res..

[34] Jiang N., Huang F., Zhang X. (2017). Smoking and the risk of diabetic nephropathy in patients with type 1 and type 2 diabetes: A meta-analysis of observational studies. Oncotarget.

[35] Wilson P.W., Kannel W.B. (2002). Obesity, diabetes, and risk of cardiovascular disease in the elderly. Am. J. Geriatr. Cardiol..

[36] Ajlouni K., Jaddou H., Batieha A. (1998). Diabetes and impaired glucose tolerance in Jordan: Prevalence and associated risk factors. J. Intern. Med..

